# Case Report: Countermeasures Against Heat and Coronavirus for Japanese Athletes at the Tokyo 2020 Olympics and Paralympic Games

**DOI:** 10.3389/fspor.2022.878022

**Published:** 2022-06-06

**Authors:** Mariko Nakamura, Takashi Naito, Tatsuya Saito, Akari Takahashi, Koji Muraishi, Noriko Hakamada, Mana Otomo, Satoshi Iizuka, Daisuke Nakamura, Hideyuki Takahashi

**Affiliations:** ^1^Department of Sports Science, Japan Institute of Sports Sciences (JISS), Tokyo, Japan; ^2^Department of Sports Research, Japan Institute of Sports Sciences (JISS), Tokyo, Japan; ^3^Faculty of Law, Hokkai-Gakuen University, Sapporo, Japan; ^4^Faculty of Medicine, Tottori University, Tottori, Japan; ^5^Faculty of Economics, Kanto Gakuen University, Ohta, Japan; ^6^Weathersnews Inc., Makuhari Techno Garden, Chiba, Japan; ^7^Faculty of Health and Sport Sciences, University of Tsukuba, Tsukuba, Japan

**Keywords:** cooling, COVID-19, elite athletes, heat acclimation, Olympics

## Abstract

The Tokyo 2020 Olympics and Paralympic Games were held in the hottest environment in the history of the games. Additionally, the worldwide coronavirus disease 2019 (COVID-19) pandemic necessitated daily polymerase chain reaction (PCR) testing during the games, wearing a mask became mandatory publicly, and it was an unheard and unique Olympic with no spectators. Heat acclimation, hydration, and body cooling are essential for safe and high-performance activities in hot environments. In 2015, the Japan Institute of Sports Sciences launched the “Heat Countermeasure Project” to conduct experiments and practical research on heat countermeasures and investigate issues related to heat countermeasures in each athletic event. The results obtained were proposed to various Japan national sports teams, and support for heat countermeasures for the Tokyo 2020 games was promoted in consultation with national federations. Furthermore, due to the COVID-19 pandemic, infectious disease countermeasures for the Tokyo 2020 Games during support were a must. Moreover, athletes, coaches, and team staff could not avoid implementing heat countermeasures while adopting measures against infectious diseases. This study aimed to clarify the issues faced with heat countermeasures and report on heat acclimation training and cooling support efforts, considering measures against infectious diseases.

## Introduction

The Tokyo 2020 Olympics and Paralympic Games (the Tokyo 2020 Games) were held in harshest heat and humid environments compared to previous summer Olympic events (Szubski, 2020). For the Tokyo 2020 Games, the International Olympics Committee Medical Department created a guidebook, “BEAT THE HEAT” (International Olympic Committee, 2020), and provided recommendations for optimizing performance and reducing the risk of heat-related illnesses. Several researchers have also recently presented data on heat countermeasures (Bongers et al., 2020; Racinais and Périard, 2020; Périard et al., 2021).

At the Japan Institute of Sports Sciences (JISS), the Heat Countermeasures Project was launched in 2015 to formulate an evidence-based body cooling strategy to support Japanese athletes in the Tokyo 2020 Games. Heat acclimation training, body cooling, and hydration reduce the risks of impaired performance and exertional heat stroke due to increased core temperature (González-Alonso et al., 1999) and dehydration (Sawka et al., 2007; Bardis et al., 2017). There are several previous studies on heat acclimation training (Chalmers et al., 2014; Racinais et al., 2015), body cooling (Racinais et al., 2015; Bongers et al., 2017; Gibson et al., 2020), and hydration (Sawka et al., 2007; Périard et al., 2021). This project aimed to organize and polish the results of these previous studies to enable coaches and athletes to easily implement them in practice and promote support for heat countermeasures in preparation for the Tokyo 2020 Games.

An additional and unique challenge for the Tokyo Games was a year of postponement due to the global coronavirus disease 2019 (COVID-19) outbreak in 2020. Organizers of The Games were required to take measures against COVID-19. This necessitated daily polymerase chain reaction (PCR) testing during games, wearing a mask became mandatory to the public, and it was an unheard and unique Olympics with no spectators. Notably, COVID-19 measures were needed to be considered during heat acclimation training, body cooling, and hydration support during the Tokyo 2020 Games, and strict adherence was required to avoid infecting others, getting infected, and generating clusters.

In this report, we aimed to clarify the issues in heat countermeasures for the Tokyo 2020 Games and report on the effects of heat acclimation training, body cooling, and hydration support considering the countermeasures against COVID-19.

## The Heat Countermeasures Project

The Heat Countermeasures Project conducted by the JISS was launched in 2015. Initially, a questionnaire survey was conducted among leading Japanese athletes and coaches (Nakamura et al., 2018), from which the following issues regarding heat countermeasures were identified. First, information was needed to be provided on effective cooling methods for exercise in the heat. Second, it was necessary to propose an effective cooling strategy that is relatively easy to implement (Nakamura et al., 2018).

Regarding the provision of information, a guidebook on heat countermeasures (Japan Institute of Sports Sciences, 2017) was published, and workshops were held in 2018 and 2019 to share up-to-date knowledge and global trends for athletes and coaches at JISS. To address the second issue, we validated the forearm cooling method and reported that a combination of forearm cooling and ice slurry ingestion resulted in a faster drop in the core temperature raised by exercise (Nakamura et al., 2020), and proposed this as a new effective and easy cooling method. Ice slurry ingestion was also found to promote a decrease in core temperature during post-exercise recovery (Nakamura et al., 2021) and decrease in brain temperature measured by non-invasive magnetic resonance spectroscopy at rest without exercise intervention (Onitsuka et al., 2018).

**Figure 1:**
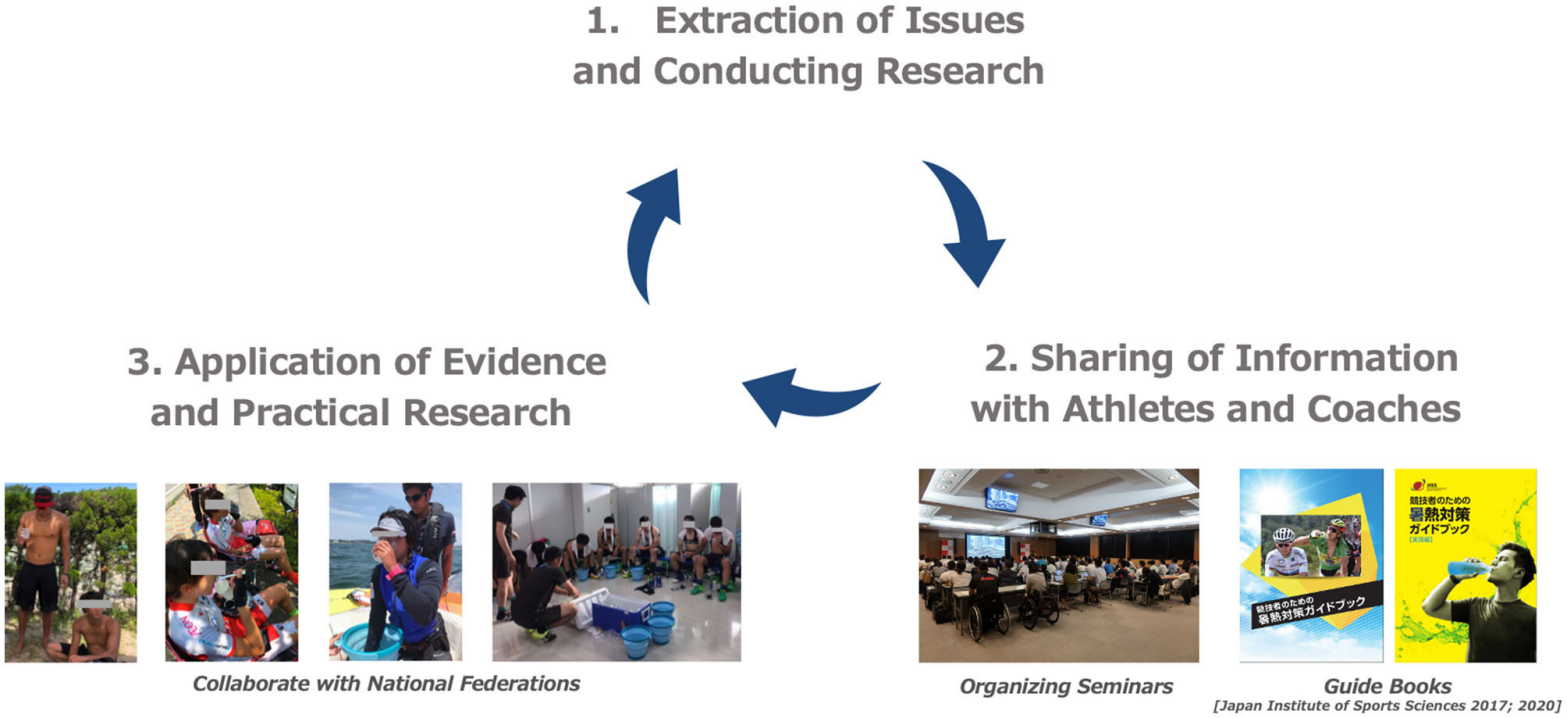
The Heat Countermeasures Project approach for the Tokyo 2020 games.

In 2019, we conducted a practical study applying evidence-based body cooling methods (Jay and Morris, 2018; Cao et al., 2022) and planned hydration for elite sailors (Nakamura et al., 2019), elite beach volleyball athletes, elite cyclists, elite fencing athletes, and the Japan national soccer team (Japan Institute of Sports Sciences, 2020). The results of the laboratory and practical studies obtained were summarized in a guidebook (Japan Institute of Sports Sciences, 2020) and provided to athletes and coaches, with pre, per, and post cooling strategies that could be implemented at the Tokyo 2020 Games. The Heat Countermeasures Project prepared for the Tokyo 2020 Games by repeating the cycle of identifying issues, conducting research, and applying evidence multiple times, as shown in Figure 1.

## Short-Term Heat Acclimation Training and Measures Against Infectious Diseases

The 3x3 basketball event, which was chosen as an official event at the Tokyo 2020 Games, was played in an outdoor court. The 3x3 Japan national team had several athletes who were also playing basketball on a five-a-side, and there were very few athletes accustomed to the outdoor hot environment. Therefore, heat acclimation training for the Tokyo 2020 Games was essential for these athletes.

Heat acclimation involving endurance training is widely known as a strategy to enhance performance in hot environments (Racinais et al., 2015). The 3x3 basketball is a type of high-intensity intermittent exercise, which required training for a limited period of 1 week for the Tokyo 2020 Games. Therefore, we focused on short-term heat acclimation training for a period of ≤ 7 days and decided to perform heat acclimation training in a hot humid chamber using a high-intensity intermittent exercise protocol (Petersen et al., 2010; Chalmers et al., 2014).

**Figure 2:**
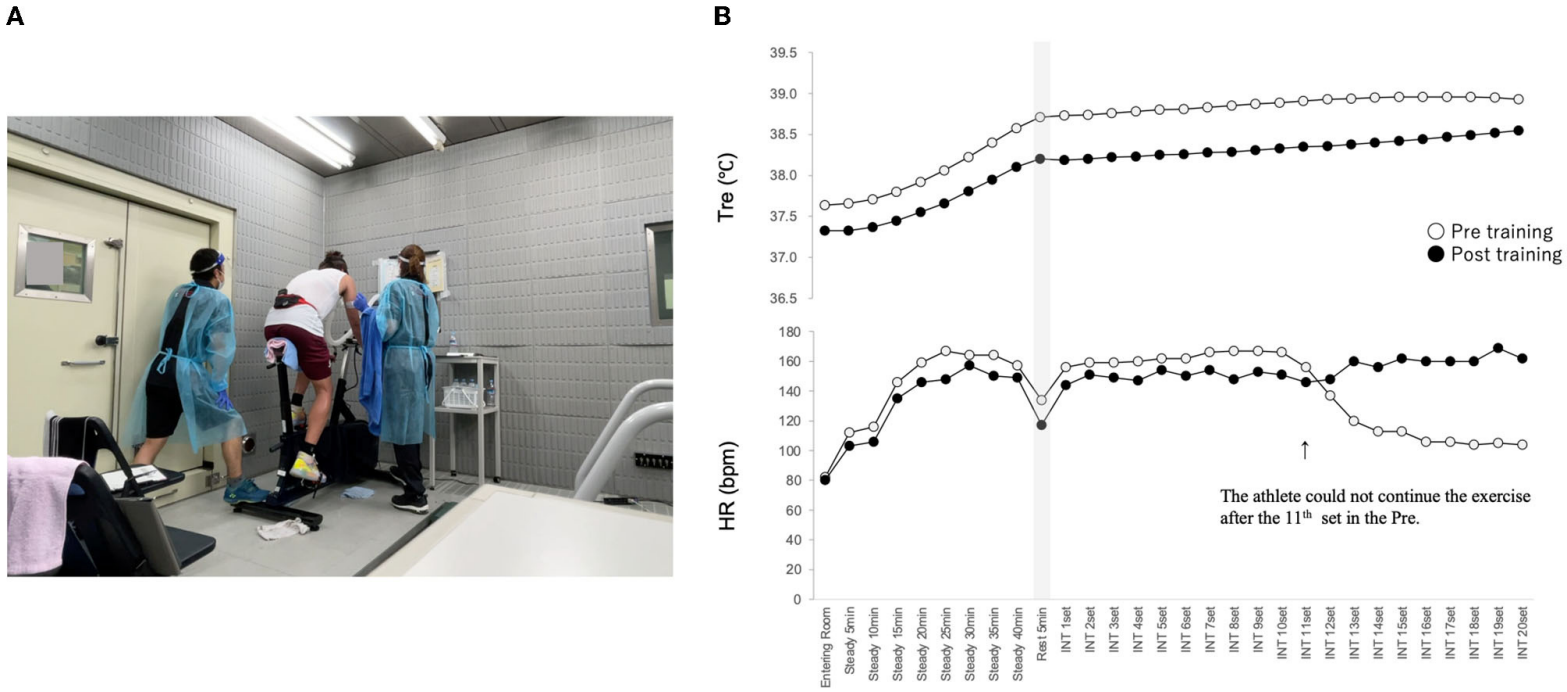
Heat acclimation training. **(A)** Heat acclimation training considering infection control. **(B)** Effects of heat acclimation training [typical core body temperature and heart rate (HR)].

COVID-19 measures were a major issue in heat acclimation training. The chamber (ESPEC Corp., Tokyo, Japan) at JISS is 4.1 × 7.2 × 2.5 m in size. Ventilation was good; however, CO_2_ levels rose to 820 ppm/h when exercising. Therefore, based on the JISS COVID-19 administration manual, we limited the number of measurers entering the room to two for each athlete. Furthermore, the athletes and researchers underwent PCR testing to confirm that they were negative for COVID-19 before starting the training. Wearing a mask during high-intensity exercise was not mandatory because it may lead to discomfort, breathing restrictions, and impaired fitness level of athletes (Al Attar and Husain, 2021). However, to prevent the risk of infection in the chamber, it was necessary to control the athlete's exhalations. Therefore, the athletes wore Rudolph masks to let their expired breath out of the room through air holes, and measures were taken to prevent CO_2_ concentration from increasing. All Rudolph masks, heart rate monitors, and thermometers were prepared for each athlete so they would not share them with other athletes. After each training session, the room was ventilated for at least 30 min, and the floor and ergometer were thoroughly disinfected. In addition, the researchers were required to wear masks, gloves, gowns, and face shields (Figure 2A).

Twelve elite athletes participated in the training. The heat acclimation training was carried out using a cycle ergometer (Fujin-Raijin, OCLabo, Tokyo, Japan) under environmental conditions of 33°C ambient temperature and 70% relative humidity. Performance tests were conducted on the 1st and 7th days. The athletes performed same load as the athletes' body weight steady-state load exercises for 10 min and 2 times body weight steady-state load exercises for 30 min. After a 5-min rest, they performed intermittent exercise in 1-min periods until exhaustion. Each period consisting of 5 s of maximal pedaling at a load of body weight × 0.075 KP, 25s of pedaling with no-work load, and 30 s of rest. Days 2–6 of training (off on day 3) consisted of a 20-min warm-up, then 1 min periods of an intermittent exercise protocol with a 5-min break for a total of 20 sets. The training time was 60 min, and the core temperature was confirmed to be above 38.5°C.

Typical data for core temperature and heart rate before and after training are shown in Figure 2B. Reduction in core temperature during the training was identified in nine of the 12 athletes before and after the training. Additionally, an improvement in the maximal and mean power output was observed in all the 12 athletes (data not shown). Although previous studies have shown that the minimum duration for partial adaptation was 8 consecutive days of exposure when the environment was hot and humid (Lei and Wang, 2021), we reported that 6 days of short-term humid heat acclimation might enhance both performance and thermoregulatory function. Throughout the training, no athlete or researchers tested positive for COVID-19.

## Body Cooling, Hydration Support, and Measures Against COVID-19

For body cooling, it is important to consider the combination of internal and external cooling (Racinais et al., 2015; Bongers et al., 2017; Jay and Morris, 2018; Gibson et al., 2020; Cao et al., 2022) and the timing of implementation (Jay and Morris, 2018; Bongers et al., 2020; Gibson et al., 2020). However, there are a number of constraints when performing body cooling during actual competitions, including individuality considerations, limitations in cooling methods and cooling times, challenges related to freezing and delivery methods of cooling devices, and venue regulation (Japan Institute of Sports Sciences, 2020; Naito et al., 2020). In addition, heat countermeasure support considering COVID-19 countermeasures was necessary at the Tokyo 2020 Games. During training and games, all support staff were also required to wear masks and gloves and to disinfect the cooling devices used. In addition, all drinks for the athletes were prepared individually for each athlete, and other infection control measures were taken according to the characteristics of each competition. Three practical examples of body cooling support for the Tokyo 2020 Games are as follows.

**Figure 3:**
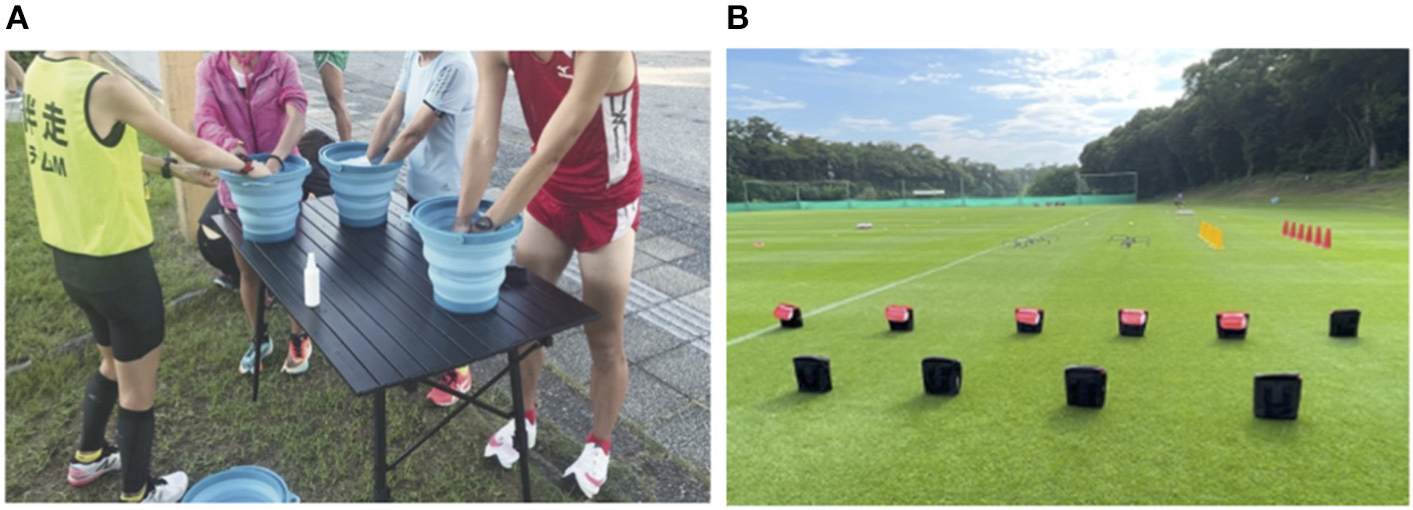
Heat and infectious disease countermeasure support for each athlete. **(A)** Forearm cooling during precooling (Takahashi et al., 2021). **(B)** Hydration measures using individual bottles.

In the blind marathon, precooling was performed by combining ice slurry ingestion with forearm cooling (Figure 3A). Ice slurry ingestion used commercial ice slurry (Pocari Sweat ice slurry, Otsuka Pharmaceuticals, Japan). This commercial ice slurry was very useful, because it was individually packaged in the prescribed amount of 100 g/piece and served as an infection control measure. In addition, forearm cooling was approached individually (Takahashi et al., 2021), and it was also useful for infection control. However, because the actual competition was held at a wet bulb globe temperature (WBGT) of 19°C, body cooling for the entire team was not implemented, but body cooling support in training for the Tokyo 2020 Games was effective.

In tennis, the combination of an ice vest and moderate intake of ice slurry was reportedly effective for athletic performance (Naito et al., 2021). This evidence helped overcome the challenges in providing cooling support at the 2020 Australian Open and involved the delivery of cooling devices through the spectator seating area (Naito et al., 2020). We made plans to bring this support to the Tokyo 2020 Games. However, the Tokyo 2020 Games were held without spectators, and support *via* spectator seats became impossible. Therefore, we prepared personal holders for the athletes in advance and distributed commercial ice slurry and ice vests for each athlete to bring to the match venue. To ensure that body cooling could be implemented during each break time, we repeated simulations from the training camp as requested by the athletes, and were able to implement it without any problems during the tournament.

In soccer, fluid intake was restricted during matches. In the case of *ad libitum* intake, performance may be reduced due to dehydration compared to prescribed intake (Bardis et al., 2017). Therefore, we implemented planned hydration to prevent impairments to the performance due to dehydration and provide the specified amount of water and ice slurry ingested per athlete's body weight during the pre-match and half-time. During training, as shown in Figure 3B, each athlete had a separate drink holder for hydration, and measures were taken to avoid sharing between athletes. In addition, forearm cooling, which involved a few athletes dipping their forearms in a bucket and sharing water, was banned because of infection control; therefore, we replaced it with ice vests. These planned hydration and body cooling support practices resulted in a dehydration rate of 2 ± 0.7% in a WBGT 26 ± 0.9°C environment throughout the six matches, which was lower than that reported in previous studies (Mohr and Krustrup, 2013; Japan Institute of Sports Sciences, 2020).

At the Tokyo 2020 Games, all of the aforementioned events that we supported won prizes. Although it is difficult to assess whether our support for heat countermeasures led to high performance and results, from the viewpoint of safety, there were no reports of poor conditions due to heat challenges from any competition or athletes, and there were no infected individuals; therefore, we suggest that the measures and support contributed to the athletes' and teams' success.

## Conclusion

It was possible to provide elite Japanese athletes and coaches with information on heat countermeasures and cooling strategies, and propose effective cooling methods for performance exertion that could be implemented in competitions. Moreover, heat acclimation training support and body cooling support considering COVID-19 countermeasures immediately before the Tokyo 2020 Games could be carried out. This case report would provide useful support resources in countermeasures for competitions in coronavirus and hot environments.

## Data Availability Statement

The original contributions presented in the study are included in the article/supplementary material, further inquiries can be directed to the corresponding author.

## Ethics Statement

The studies involving human participants were reviewed and approved by Ethics Committee of the Japan Institute of Sports Sciences. The patients/participants provided their written informed consent to participate in this study.

## Author Contributions

MN contributed to the management and oversee projects, data collection, research, support, manuscript writing, and review process. TN contributed to the planning of the project, data collection, research, support, and as well as critical of the manuscript. TS, AT, KM, NH, MO, and SI contributed to the acquisition, analysis, interpretation of the data, and support for elite athletes. DN contributed to the launch of the project and was involved in the project's planning, data collection, research, and support. HT contributed to the launch of the project and critical revisions of the manuscript. All authors contributed to manuscript revision, read, and approved the submitted version.

## Conflict of Interest

DN was employed by Weathersnews Inc. The remaining authors declare that the research was conducted in the absence of any commercial or financial relationships that could be construed as a potential conflict of interest.

## Publisher's Note

All claims expressed in this article are solely those of the authors and do not necessarily represent those of their affiliated organizations, or those of the publisher, the editors and the reviewers. Any product that may be evaluated in this article, or claim that may be made by its manufacturer, is not guaranteed or endorsed by the publisher.

## References

[B1] Al Attar W. S. A. Husain M. A. (2021). Does exercising with a face mask affect athletes performance? Br. J. Sports Med. 55:A163. 10.1136/bjsports-2021-IOC.392

[B2] Bardis C. N. Kavouras S. A. Adams J. D. Geladas N. D. Panagiotakos D. B. Sidossis L. S. (2017). Prescribed drinking leads to better cycling performance than ad libitum drinking. Med. Sci. Sports Exerc. 49, 1244–1251. 10.1249/MSS.0000000000001202 28079705

[B3] Bongers C. C. de Korte J. Q. Eijsvogels T. (2020). Infographic. Keep it cool and beat the heat: cooling strategies for exercise in hot and humid conditions. Br. J. Sports Med. 55, 643–644. 10.1136/bjsports-2020-102294 32561517

[B4] Bongers C. C. Hopman M. T. Eijsvogels T. M. (2017). Cooling interventions for athletes: an overview of effectiveness, physiological mechanisms, and practical considerations. Temperature 4, 60–78. 10.1080/23328940.2016.1277003 28349095 PMC5356217

[B5] Cao Y. Lei T. H. Wang F. Yang B. Mündel T. (2022). Head, face and neck cooling as per-cooling (cooling during exercise) modalities to improve exercise performance in the heat: a narrative review and practical applications. Sports Med. Open 8:16. 10.1186/s40798-022-00411-4 35092517 PMC8800980

[B6] Chalmers S. Esterman A. Eston R. Bowering K. J. Norton K. (2014). Short-term heat acclimation training improves physical performance: a systematic review, and exploration of physiological adaptations and application for team sports. Sports Med. 44, 971–988. 10.1007/s40279-014-0178-6 24817609

[B7] Gibson O. R. James C. A. Mee J. A. Willmott A. G. B. Turner G. Hayes M. (2020). Heat alleviation strategies for athletic performance: a review and practitioner guidelines. Temperature 7, 3–36. 10.1080/23328940.2019.1666624 32166103 PMC7053966

[B8] González-Alonso J. Teller C. Andersen S. L. Jensen F. B. Hyldig T. Nielsen B. (1999). Influence of body temperature on the development of fatigue during prolonged exercise in the heat. J. Appl. Physiol. 86, 1032–1039. 10.1152/jappl.1999.86.3.1032 10066720

[B9] International Olympic Committee (2020). Beat the Heat at Tokyo 2020. International Olympic Committee. Available online at: https://www.olympic.org/athlete365/games-time/beat-the-heat/ (accessed January 22, 2022).

[B10] Japan Institute of Sports Sciences (2017). Guidebook for Athletes on Heat Countermeasures [Basic]. Tokyo: High Performance Sports Center, Japan Institute of Sports Sciences.

[B11] Japan Institute of Sports Sciences (2020). Guidebook for Athletes on Heat Countermeasures [Practice]. Tokyo: High Performance Sports Center, Japan Institute of Sports Sciences.

[B12] Jay O. Morris N. B. (2018). Does cold water or ice slurry ingestion during exercise elicit a net body booling effect in the heat? Sports Med. 48, 17–29. 10.1007/s40279-017-0842-8 29368184 PMC5790850

[B13] Lei T. H. Wang F. (2021). Looking ahead of 2021 Tokyo Summer Olympic Games: How does humid heat affect endurance performance? Insight into physiological mechanism and heat-related illness prevention strategies. J. Therm. Biol. 99:102975. 10.1016/j.jtherbio.2021.102975 34420619

[B14] Mohr M. Krustrup P. (2013). Heat stress impairs repeated jump ability after competitive elite soccer games. J. Strength Cond. Res. 27, 683–689. 10.1519/JSC.0b013e31825c3266 23443218

[B15] Naito T. Nakamura M. Muraishi K. Eda N. Ando K. Takemura A.. (2021). In-play optimal cooling for outdoor match-play tennis in the heat. Eur. J. Sport Sci. 18, 1–10. 10.1080/17461391.2020.1870160 33393422

[B16] Naito T. Saito T. Tajima T. Someya S. Tsuchihashi T. (2020). The challenges and strategies of support using body cooling in tennis competition: a case report of support in 2020 Australian Open. J. High Perform. Sport 6, 118–128. 10.32155/jissjhps.6.0_118

[B17] Nakamura D. Hasegawa H. Nakamura M. Hagiwara M. Takahashi H. (2019). Effect of cooling strategy on physiological and perspective response of elite woman sailing athlete during exercise in warm environment: a case study. J. High Perform. Sport 4, 145–153. 10.32155/jissjhps.4.0_145

[B18] Nakamura D. Muraishi K. Hasegawa H. Yasumatsu M. Takahashi H. (2020). Effect of a cooling strategy combining forearm water immersion and a low dose of ice slurry ingestion on physiological response and subsequent exercise performance in the heat. J. Therm. Biol. 89:102530. 10.1016/j.jtherbio.2020.102530 32364976

[B19] Nakamura D. Tanabe Y. Takahashi H. (2018). Cooling strategies of elite Japanese athletes. A questionnaire-based study. Sports Sci. Elite Athlete Sup. 3, 39–51. 10.32155/jiss.3.0_39

[B20] Nakamura M. Nakamura D. Yasumatsu M. Takahashi H. (2021). Effect of ice slurry ingestion on core temperature and blood pressure response after exercise in a hot environment. J. Therm. Biol. 98:102922. 10.1016/j.jtherbio.2021.102922 34016346

[B21] Onitsuka S. Nakamura D. Onishi T. Arimitsu T. Takahashi H. Hasegawa H. (2018). Ice slurry ingestion reduces human brain temperature measured using non-invasive magnetic resonance spectroscopy. Sci. Rep. 8:2757. 10.1038/s41598-018-21086-6 29426888 PMC5807509

[B22] Périard J. D. Eijsvogels T. Daanen H. A. M. Racinais S. (2021). Hydration for the Tokyo Olympics: to thirst or not to thirst? Br. J. Sports Med. 55, 410–411. 10.1136/bjsports-2020-102803 32883691

[B23] Petersen C. J. Portus M. R. Pyne D. B. Dawson B. T. Cramer M. N. Kellett A. D. (2010). Partial heat acclimation in cricketers using a 4-day high intensity cycling protocol. Int. J. Sports Physiol. Perform. 5, 535–545. 10.1123/ijspp.5.4.535 21266737

[B24] Racinais S. Alonso J. M. Coutts A. J. Flouris A. D. Girard O. González-Alonso J. (2015). Consensus recommendations on training and competing in the heat. Br. J. Sports Med. 49, 1164–1173. 10.1136/bjsports-2015-094915 26069301 PMC4602249

[B25] Racinais S. Périard J. D. (2020). Benefits of heat re-acclimation in the lead-up to the Tokyo Olympics. Br. J. Sports Med. 54, 945–946. 10.1136/bjsports-2020-102299 32276924

[B26] Sawka M. N. Burke L. M. Eichner E. R. Maughan R. J. Montain S. J. Stachenfeld N. S. (2007). American College of Sports Medicine position stand. Exercise and fluid replacement. Med. Sci. Sports Exerc. 39, 377–390. 10.1249/mss.0b013e31802ca597 17277604

[B27] Szubski C.. (2020). Sweltering Heat at the 2020 Olympics in Tokyo. Available online at: https://sportifycities.com/tokyo-2020-heat-factor/ (accessed February 2, 2022).

[B28] Takahashi A. Muraishi K. Hakamada N. Nakamura M. (2021). Heat countermeasure support for blind marathon athletes for the 2020 Tokyo Paralympic Games. J. High Perform. Sport 7, 51–57. 10.32155/jissjhps.7.0_51

